# The knowns and unknowns of perfusion disturbances in COVID-19 pneumonia

**DOI:** 10.1186/s13054-021-03742-y

**Published:** 2021-09-28

**Authors:** Mattia Busana, Lorenzo Giosa

**Affiliations:** 1 Department of Anesthesiology, University Medical Center Göttingen, Robert Koch Straße 40, 37075 Göttingen, Germany; 2 Department of Surgical Sciences, University of Turin, Turin, Italy

**Editor,**

Ball et al. [1] recently proposed a thoughtful quantitative analysis of gas and blood distribution in the lungs of patients with severe COVID-19 pneumonia. The paper offers some interesting cues that we consider worth discussing.

First, the images of dual-energy CT scan (DECT) were used as surrogates of the ventilation and perfusion distribution in the lung. However, other imaging techniques, like scintigraphy and SPECT, might be more suitable for this task. Indeed, while perfusion is likely accurately depicted by DECT, *ventilation* is not. Indeed, for at least two reasons, the gas content measured by this technique (notably during tidal breathing and not at constant airway pressure) may not represent a good surrogate of the distribution of ventilation:

1. Airway closure may dissociate the instantaneous gas volume from the actual ventilation, and the phenomenon may be more common than currently thought [2];
2. The opening pressures cannot be investigated by a single CT scan [3]. It follows that undetected recruitability (possibly nonlinear along the pressure–volume curve) may contribute to the uncoupling between measured gas volume and alveolar ventilation.

This leads, in our opinion, to some inconsistencies. Most importantly, in Fig. 4, the authors show a plot reasonably inspired by the V_A_/Q_T_ distributions obtained with the multiple inert gas elimination technique. Unfortunately, such distribution is likely far from what we may expect from COVID-19 pneumonia. Indeed, the displayed distributions are essentially centered on a gas–blood volume ratio ~ 1. However, as we have shown in our theoretical model [4], such distribution (coupled with the relatively low shunt fraction reported in Table 2) is not compatible with the severe hypoxemia observed in these patients.

As a side note, considering that the analysis performed by Ball et al. excluded large vessels, one may wonder how the presence of abnormal vasodilation and vascular anastomoses [5] would affect their results. Indeed, *nonfunctional* vessels in a “gas-rich” region may escape the definition of shunt used by the authors.

## Conclusions

The impact of perfusion alterations on gas exchange in COVID-19 is far from being understood. Despite the aforementioned limitations, Ball et al. must be congratulated for their effort in reporting compelling data that represent a smart step forward in the understanding and, most importantly, in the quantification of the problem.

## Data Availability

NA.

## Abbreviations

DECT: Dual-energy CT scan
SPECT: Single photon emission computed tomography
V_A_/Q_T_: Ventilation–perfusion distribution
