# Supplementary material for: The knowns and unknowns of perfusion disturbances in COVID-19 pneumonia
Source: Crit Care. 2021 Sep 28;25:352. doi: 10.1186/s13054-021-03742-y (PMC8476978; doi:10.1186/s13054-021-03742-y)
Supplement: Related texts

# Reply to: The knowns and unknowns of perfusion disturbances in COVID-19 pneumonia

**Authors:** Lorenzo Ball, Chiara Robba, Jacob Herrmann, Sarah E. Gerard, Yi Xin, Maura Mandelli, Denise Battaglini, Iole Brunetti, Giuseppe Minetti, Sara Seitun, Giulio Bovio, Antonio Vena, Daniele Roberto Giacobbe, Matteo Bassetti, Patricia R. M. Rocco, Maurizio Cereda, Rahim R. Rizi, Lucio Castellan, Nicolò Patroniti, Paolo Pelosi

We would like to thank Dr. Busana and Dr. Giosa for their interest in our study [1] and for giving us the opportunity to expand the discussion regarding disturbances of lung perfusion in COVID-19 patients. They highlighted the differences between ventilation and static aeration as assessed by CT; questioned the interpretation of our gas:blood ratio distribution plots and the definition of shunt, comparing our in vivo results with those of their computational theoretical model [4]. As per local practice during the COVID-19 pandemic, CT scans were acquired during breath-hold and we used lung aeration to define shunt, dead space, and non-aerated/non-perfused areas. Lung aeration may be considered as a potential surrogate of ventilation, especially when protective tidal volume and limited inspiratory plateau pressure are used, as in the case of COVID-19 mechanically ventilated patients [6]. In this context, it seems reasonable to assume that non-aerated areas are also non-ventilated and that aerated regions receive a certain amount of ventilation, especially given the small extent of hyper-aeration and poor recruitment with application of PEEP observed in these patients [7]. The pulmonary gas:blood volume ratio was defined as:

$${\text{Gas}}{:}\;{\text{Blood}}\;{\text{volume}}\;{\text{ratio}} = \frac{{f_{{\text{A}}} \cdot \overline{{{\text{PBV}}}} }}{{{\text{PBV}} \cdot \overline{{f_{{\text{A}}} }} }}$$

namely as the ratio of mean-normalized gas fraction to mean-normalized pulmonary blood volume: based on this definition, the values of the ratio are centered around 1 in each patient and cannot be directly translated into V′_A_/Q′_T_ without knowing the cardiac output and alveolar minute ventilation. Such a ratio allows within-patient comparisons of the relative distribution of aeration and perfusion. We excluded only large lung vessels from the analysis: the vasodilation of peripheral vessels affects V′_A_/Q′_T_ at an anatomical scale well below the size of one CT voxel [8]: voxels comprising dilated vessels and patent alveoli would have an intermediate CT attenuation value and a high pulmonary blood volume, corresponding to our “*gas:blood ratio* < *1*” compartment.

Despite several technical limitations of the technique we proposed, our overall interpretation of the data is that in the early stages of the disease, hypoxia is explained by low V′_A_/Q′_T_ responsive to higher inspiratory oxygen fractions and non-invasive respiratory assistance, while also by increased true shunt in the late stages requiring invasive mechanical ventilation.

In conclusion, we believe that our data should not be interpreted as in contrast with your theoretical model, but rather could represent its confirmation and an attempt of explaining in vivo gas-exchange changes in critically ill patients with severe COVID-19 pneumonia.
